# Comparative Quantitative Proteomic Analysis of High and Low Toxin-Producing *Karenia brevis* Strains Reveals Differences in Polyketide Synthase Abundance and Redox Status of the Proteome

**DOI:** 10.3390/md23070291

**Published:** 2025-07-17

**Authors:** Kathleen S. Rein, Ricardo Colon, Carlos R. Romagosa, Nicholas R. Ohnikian, Kirstie T. Francis, Samuel R. Rein

**Affiliations:** 1 Department of Marine and Earth Science, The Water School, Florida Gulf Coast University, Fort Myers, FL 33965, USA; 2 Department of Chemistry and Biochemistry, Florida International University, Miami, FL 33199, USA; ricky.colon0794@gmail.com (R.C.); croma056@fiu.edu (C.R.R.); 3 Mote Marine Laboratory and Aquarium, Sarasota, FL 34236, USA; nohnikian@mote.org (N.R.O.); kfrancis@mote.org (K.T.F.); 4 The School District of Philadelphia, Philadelphia, PA 19130, USA

**Keywords:** *Karenia brevis*, proteomics, polyketide synthase, brevetoxin

## Abstract

To identify differentially abundant polyketide synthases (PKSs) and to characterize the biochemical consequences of brevetoxin biosynthesis, bottom-up, TMT-based quantitative proteomics and redox proteomics were conducted to compare two strains of the Florida red tide dinoflagellate *Karenia brevis*, which differ significantly in their brevetoxin content. Forty-eight PKS enzymes potentially linked to brevetoxin production were identified, with thirty-eight showing up to 16-fold higher abundance in the high-toxin strain. A pronounced shift toward a more oxidized redox state was observed in this strain’s proteome. Notably, 25 antioxidant-related proteins were significantly elevated, including alternative oxidase (AOX), which increased by 17-fold. These results elucidate the cellular consequences of toxin biosynthesis in *K. brevis*, offer new leads for the study of brevetoxin biosynthesis, and suggest a novel red tide mitigation approach targeting high toxin-producing strains.

## 1. Introduction

**Figure 1:**
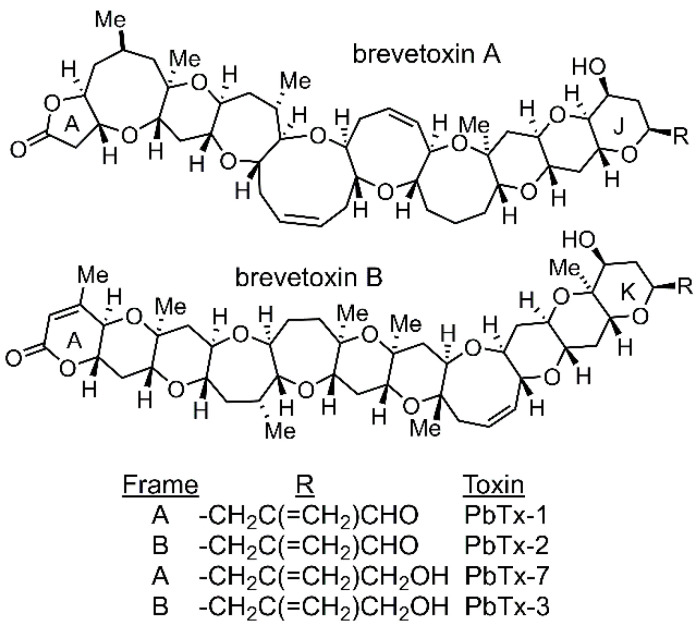
Structures of the brevetoxins.

Recurring blooms of *Karenia* brevis, the Florida red tide dinoflagellate, in the Gulf of Mexico, are a bane to the shellfish and tourism industries in the region and can precipitate environmental, economic, and public health-related catastrophes. Extended events can persist for months or even years [1], resulting in massive wildlife mortalities [2] and economic losses for the shellfish and tourism industries [3,4]. Humans exposed to the Florida red tide may experience respiratory, gastrointestinal, or neurological disorders [5,6] through the inhalation of toxic aerosols or the consumption of tainted shellfish. These harmful effects are caused by a suite of neurotoxins, known as brevetoxins, which are produced by *K. brevis*. The brevetoxins belong to a small class of secondary metabolites called polyether ladders (Figure 1). More than a dozen structural variants of the brevetoxins have been reported to date [7]. However, by far the most abundant of the brevetoxins is PbTx-2 [7,8].

In higher organisms, brevetoxins activate the voltage-gated sodium channel (VGSC), affecting both the central and peripheral nervous systems as well as skeletal muscle [9]. Yet, the endogenous function of brevetoxins remains uncertain. It has been suggested that brevetoxins act as a deterrent to grazing by predators [10,11,12] or that they play an allelopathic role, allowing *K. brevis* to outperform phytoplankton competitors [13,14,15,16,17]. The localization of the brevetoxins to the chloroplast of *K. brevis* using a fluorescent brevetoxin derivative and the capture of two chloroplast proteins using a brevetoxin photoaffinity probe (the light harvesting complex II (LHC-II) and a thioredoxin (Trx)-like protein [18]) suggested a role in photosynthetic processes and/or regulation of redox status of the cell. It was later demonstrated that PbTx-2 is an inhibitor of recombinant *K. brevis* thioredoxin reductase *Kb*TrxR [19].

Precursor incorporation experiments have established the polyketide origins of brevetoxins [20]. Over the past three decades, genes encoding hundreds of polyketide biosynthetic pathways in bacteria, plants, and fungi have been identified, cloned, and heterologously expressed [21,22]. Such studies have expanded our understanding of these pathways and facilitated the production of valuable polyketide products [23] to include novel structures resulting from genetic alterations and recombinations [24]. Despite these advances, peculiarities of the dinoflagellate genome and their mechanisms for regulating gene expression have made the analysis of polyketide biosynthesis in dinoflagellates at the genome level thus far impenetrable. Dinoflagellates have large, complex genomes characterized by repetitive tandem gene arrays and a high abundance of modified nucleotides [25]. RNA trans-splicing is common in dinoflagellates [26,27,28], and gene expression appears to be regulated largely at the translational or post-translational level rather than at the transcriptional level [29]. While a handful of reports describe the transformation of dinoflagellate chloroplast [30] or nuclear genomes [31,32,33], no robust method currently exists for the genetic transformation of dinoflagellates, in particular for those with large repetitive genomes. Due in part to these limitations, much effort has focused on de novo transcriptome assembly to catalog dinoflagellate-derived polyketide synthase (PKS) transcripts that may encode the biosynthetic enzymes involved in these pathways [34,35,36]. Such studies have revealed an unexpectedly large number of transcripts, many of which do not conform to predicted architectures based on toxin structures, and few correlations between transcript abundance and toxin content have emerged.

While non-toxic strains of *K. brevis* have not been identified, cellular toxin loads can vary widely in *K. brevis* blooms, ranging from less than 1 up to 68 pg/cell [37,38]. Laboratory strains of *K. brevis* have been reported to have sustained differences in toxin content by up to 10-fold [8,39]. Two strains, both derived from the same (Wilson) strain of *K. brevis*, inexplicably have shown a sustained 10-fold difference in brevetoxin content for over a decade. The availability of high (*Kb*HT) and low (*Kb*LT) toxin-producing strains of *K. brevis* provides an outstanding opportunity to probe the endogenous function of the brevetoxins as well as the pathway for their biosynthesis. We hypothesize that the proteomes of *Kb*HT and *Kb*LT will differ significantly in enzymes related to brevetoxin biosynthesis and that the proteomes will be altered due to the biochemical effects of brevetoxins. In this work, we have compared the log-phase cultures of *Kb*HT and *Kb*LT by bottom-up quantitative proteomics using amine reactive tandem mass tags (TMT) to identify differentially abundant proteins (DAPs) that may be related to brevetoxin biosynthesis. Furthermore, if PbTx-2 is indeed a regulator of cellular redox status, then these two strains should exhibit discernable differences in the redox state of their proteomes, which would be reflected by the redox state of the protein cysteine residues. Hence, we have performed redox proteomics utilizing cysteine reactive iodo-TMT reagents to identify and quantify those differences. Log-phase cultures were chosen as they are likely to exhibit higher metabolic activity when compared to stationary phase cultures. Van Dolah and co-workers found more multimodule PKS transcripts using log-phase cultures of the ciguatoxin producing *Gambierdiscus polynesiensis* when comparing non-toxic *G. pacificus* [40]. Furthermore, Monroe, et al. reported higher levels of two PKS proteins in *Kb*HT compared to *Kb*LT by Western blot of log-phase cultures [41]. Our studies have revealed differentially abundant proteins that may be related to toxin biosynthesis and global disparities in the redox states of the two *K. brevis* proteomes.

## 2. Results

### 2.1. Differences in Redox Stoichiometry Between High- and Low-Toxin K. brevis

**Figure 2:**
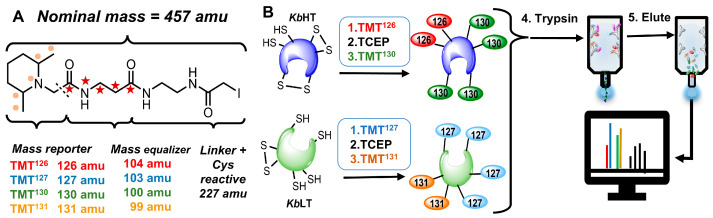
(**A**) Isobaric iodo-TMT cysteine-labeling reagent. The mass of the reporter is balanced by the mass equalizer such that each label has the same nominal mass. Locations of labels are shown for TMT131 (●) and TMT126(★). (**B**) Redox proteomics workflow. 1. Cysteine residues were labeled with the light label (iodoTMT126 and iodoTMT127 for *Kb*HT and *Kb*LT, respectively). 2. Unlabeled cysteine residues were reduced with TCEP. 3. Heavy tags (iodoTMT130 and iodoTMT131 for *Kb*HT and *Kb*LT, respectively) were used to label newly reduced cysteine residues. 4. Equal amounts of labeled proteins (according to Bradford assay) were combined, trypsinized, and purified using Immobilized anti-TMT resin (black triangles represent unlabeled peptides which will not bind to the resin). 5. Purified, labeled peptides were eluted from the resin and analyzed via LC-MS/MS. Figure modified from Colon and Rein [46].

Iodo-TMT reagents are useful for analyzing the redox status of cysteine residues in proteins [42,43]. Like amine reactive TMTs, iodo-TMTs have five isotopic labels (either 15N or 13C: see Figure 2A) distributed across the mass reporter and the mass equalizer, such that the total nominal mass is the same for all reagents, but each reporter will have a unique mass. Using the differential cysteine-labeling strategy [44,45] outlined in Figure 2B, the redox stoichiometry of individual proteins was obtained from the ratio of reduced to total (TMT_red_/TMT_total_) cysteines.

**Figure 3:**
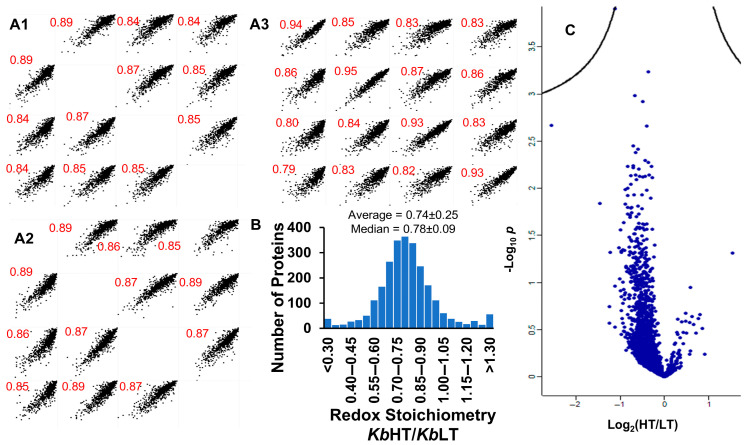
(**A**) Multi-scatter plots of log-transformed ratio of redox stoichiometry for (**A1**) *Kb*HT, (**A2**) *Kb*LT, and (**A3**) *Kb*HT/*Kb*LT. (**B**) Histogram of fold change of redox state for *Kb*HT/*Kb*LT for all proteins identified. (**C**) Volcano plot of log_2_ of fold change of redox state for *Kb*HT/*Kb*LT. (FDR 0.05).

In total, 2542 proteins were identified among four replicate samples. Among these 2542 proteins, 1092 occurred in three or more replicates. To assess reproducibility, multi-scatter plots of biological replicate samples were generated and are shown in Figure 3(A1–A3). Pearson correlation coefficients ranged from 0.85 to 0.89 for both *Kb*HT and *Kb*LT indicating good reproducibility across replicates. A histogram of the fold change of redox stoichiometry (TMT_red_/TMT_total_) for all 2542 proteins for *Kb*HT/*Kb*LT is shown in Figure 3B. Cysteine residues were significantly more oxidized (lower redox stoichiometry) in *Kb*HT. The average redox stoichiometries were 0.28 ± 0.15 and 0.37 ± 0.19 (*p* = 0, two-tailed paired *t*-test) for *Kb*HT and *Kb*LT, respectively. Shown in Figure 3B, the average and median values for the fold change of redox stoichiometry *Kb*HT/*Kb*LT were 0.74 ± 0.25 and 0.78 ± 0.09, respectively. The shift in the ratios of redox stoichiometry for *Kb*HT/*Kb*LT towards values less than one is indicative of a more oxidized proteome in *Kb*HT. A volcano plot of the redox stoichiometry of all proteins is shown in Figure 3C. While 85% of the proteins showed a reduced redox stoichiometry in *Kb*HT, differences for individual proteins were not significant when applying a false discovery rate (FDR) of 0.05. Hierarchical clustering failed to reveal any discernible clusters in the data. Among the 1092 proteins that appeared in three or more replicates, 758 could be annotated with confidence and assigned COG (Clusters of Orthologous Groups) [47] terms. Classifying proteins into COG terms provided a functional framework for interpreting the proteomic changes observed. This allowed us to identify which biological processes or pathways, if any, were most altered between conditions. Fisher’s exact test for the enrichment of COG terms after filtering out unannotated proteins did not identify any significant associations. A gene set enrichment analysis (GSEA) of redox stoichiometry, performed by submitting a ranked list (fold change) of gene names to WebGestalt [48] against *Arabidopsis thaliana*, did not return any significantly enriched pathways. The algorithm DeepLoc 2.1 [49] was applied to assign all 2542 proteins into 10 cellular compartments. Fisher’s exact test was performed to assess the association between redox stoichiometry and cellular compartments, but no significant associations were identified.

### 2.2. Differentially Abundant Proteins Between High- and Low-Toxin K. brevis

**Figure 4:**
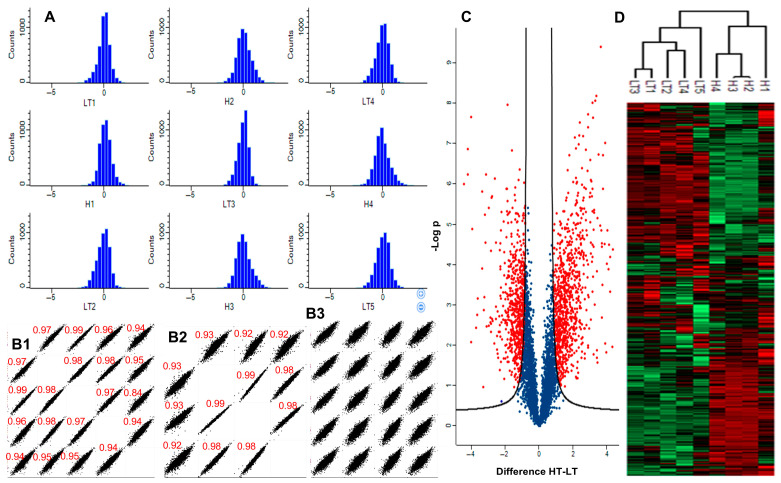
(**A**) Log-intensity histograms for *Kb*HT and *Kb*LT samples normalized to the mean. (**B**) Multi-scatter plots of pairwise log-intensity distribution for all samples: (**B1**) *Kb*LT (Pearson correlation coefficients ≥ 0.94), (**B2**) *Kb*HT (Pearson correlation coefficients ≥ 0.91), and (**B3**) *Kb*HT/*Kb*LT (Pearson correlation coefficients > 0.85). (**C**) Volcano plot of log_2_ fold change *Kb*HT/*Kb*LT vs. -log_10_*p*. (FDR 0.02, S 1.0) DAPs are red and non-significant proteins are blue. (**D**) Heat map showing hierarchical clustering for all identified proteins. Red and green colors represent more and less abundant proteins, respectively.

Proteins were analyzed from four biological replicate samples of *Kb*HT and five biological replicate samples of *Kb*LT. An LC-MS/MS analysis identified 5044 unique proteins of which only 2061 could be assigned a function and 1925 assigned a COG classification. Log-intensity histograms, after scaling channels to the same intensity and normalization to the mean, are shown for all nine samples in Figure 4A and demonstrate normal distributions and consistent intensities across replicates. Multi-correlation plots are shown in Figure 4B. Pearson correlation coefficients ranged from 0.92 to 0.99 for *Kb*HT and 0.94 to 0.99 for *Kb*LT, indicating excellent reproducibility within biological replicates. Pearson correlation coefficients for *Kb*HT vs. *Kb*LT ranged from 0.85 to 0.93, indicating slightly more variability between the strains. As shown in the volcano plot in Figure 4C, 1910 proteins were identified as DAPs (FDR = 0.02, S = 1) with 859 more abundant in *Kb*HT. A hierarchical clustering analysis based on protein expression profiles resulted in the identification of two distinct clusters that reflected the expected groupings of *Kb*HT and *Kb*LT (Figure 4D).

**Figure 5:**
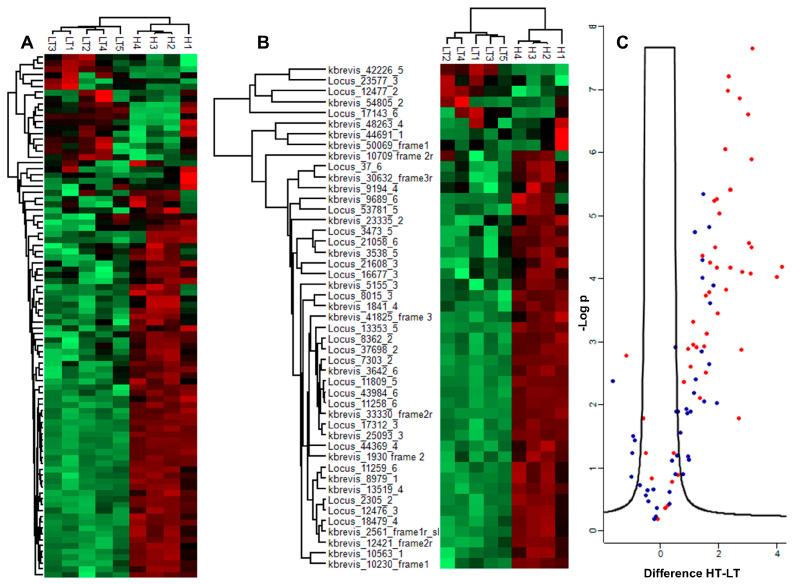
(**A**) Heat map of COG Q (secondary metabolite biosynthesis). (**B**) Heat map of COG Q PKS (polyketide synthase). Red and green colors on the heat maps represent more and less abundant proteins, respectively. Names starting with “Locus” are translated from [56]. Names starting with “kbrevis” are translated from [34]. The names end in the frame of translation. (**C**) Volcano plot of log_2_ fold change *Kb*HT/*Kb*LT vs. −log_10_*p* (FDR 0.02, S 1.0) COG Q shown in blue PKS shown in red. Heat map 5A with all protein Loci may be found in Supplemental File S5.

Among the 1910 proteins that were significantly altered, only 675 could be assigned COG terms. After filtering for annotated proteins, Fisher’s exact test revealed significant associations with three COG term categorical annotations: A (RNA processing and modification, enrichment *Kb*HT/*Kb*LT = 0.50, *p-*value = 1.6 × 10^−5^, FDR = 6.4 × 10^−3^), T (signal transduction mechanisms, enrichment *Kb*HT/*Kb*LT =0.65, *p-*value = 5.8 × 10^−4^, FDR = 1.6 × 10^−2^), and Q (secondary metabolite biosynthesis, enrichment *Kb*HT/*Kb*LT = 1.54, *p-*value = 1.02 × 10^−6^, FDR = 8.02 × 10^−5^). Enrichment scores indicate more uniformity across COG terms A and T and enhanced variability across COG term Q. Within the category of secondary metabolite biosynthesis, 48 proteins were annotated as polyketide synthases. Fisher’s exact test on this subset of Q, which we call “Q PKS”, revealed an even more significant association (enrichment *Kb*HT/*Kb*LT = 1.81, *p-*value = 1.0 × 10^−7^, FDR = 8.1 × 10^−6^). Heat maps of COG Q and Q PKS are shown in Figure 5A,B respectively. The 1D annotation enrichment feature of the Perseus software determined whether the proteins tended to be ranked higher (or lower) by categorical class between the two observation groups [50]. Comparing the normalized intensity of each protein, this analysis determined that proteins belonging to secondary metabolite biosynthesis (COG Q) and to polyketide biosynthesis (Q PKS) were higher in all four *Kb*HT replicates (*p-*values < 2.1 × 10^−11^, FDR < 1.7 × 10^−9^) and lower in all five *Kb*LT replicates (*p-*values < 2.3 × 10^−11^, FDR < 1.9 × 10^−9^). Ranking all DAPs by fold change revealed that the protein with the fourth highest fold change and the first that was annotated was a PKS (16-fold). Among the 48 PKS proteins identified in our dataset, 39 differed significantly between the two strains, with 38 having a fold change >1 (1.7 to 18) and one <1 (0.45) for *Kb*HT/*Kb*LT. These 48 PKS proteins included five multidomain PKSs corresponding to those reported at the transcript level by Van Dolah [34]. Four of the multidomain PKSs found in our dataset were more abundant in *Kb*HT having a fold change >1 (1.3 to 6.9), and three of the five were significantly more abundant in *Kb*HT. The remaining 43 PKSs in our dataset were listed as single-domain enzymes, while two PKSs consisted of two or three repeated KS domains. We confirmed the presence of acyl carrier protein (ACP), keto-synthase (KS), dehydratase (DH), and keto-reductase (KR) domains in these PKSs by conducting our own sequence and structure-based analyses using SBSPKS v2, the Search PKS/NRPS tool, and InterProScan [51,52,53]. Signal peptides which would target the proteins to the plastid were identified in three of our PKS proteins, but these three were not significantly different between *Kb*HT and *Kb*LT. All other PKS proteins in our dataset were localized to the cytoplasm by the DeepLoc algorithm. Interestingly, Monroe and co-workers were able to localize one PKS to the plastid [41]. Of note is the identification of a trans-acting enoyl reductase (ER), which was grouped into COG I (fatty acid biosynthesis and metabolism) among the DAPs that exhibited a 12-fold increase for *Kb*HT/*Kb*LT. Not only are trans-acting ERs involved with the biosynthesis of polyunsaturated fatty acids in algae [54], but also polyketide biosynthesis in fungi [55].

Twenty-seven of the PKSs identified in our dataset are common with those reported by Van Dolah. These PKSs were placed within a monophyletic clade of protist PKSs by phylogenetic analysis [34]. Among those unique to this study, a BLASTp [57] search against the NCBI bacteria (Taxid: 2) and the SAR supergroup (taxid:2698737) databases was performed. The distance tree generated from the top-scoring hits (via NCBI’s default Fast Minimum Evolution method) revealed that the query PKSs grouped exclusively within a clade of dinoflagellate sequences. Notably, no close relationship was observed with bacterial sequences, which formed a separate and distinct branch.

**Figure 6:**
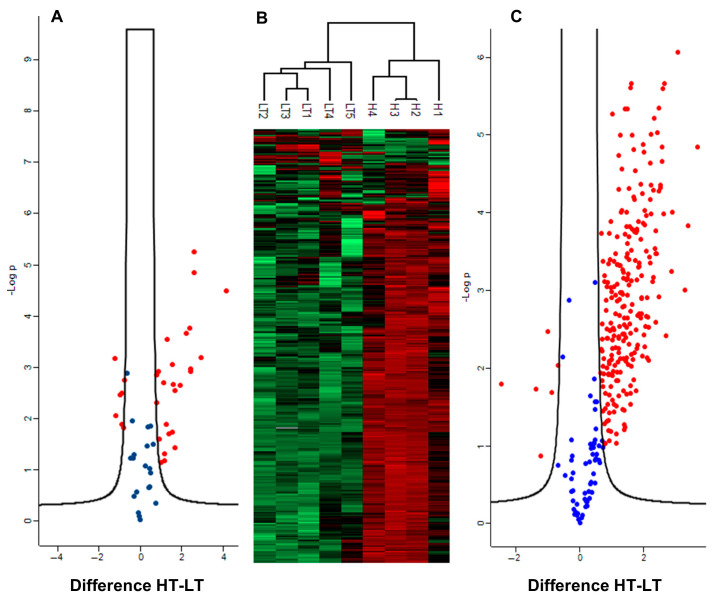
(**A**) Volcano plot of 52 proteins related to antioxidant defense. (**B**) Heat map showing hierarchical clustering of *Kb*HT and *Kb*LT plastid proteins. Red and green colors on the heat map represent more and less abundant proteins, respectively. (**C**) Volcano plot of all proteins localized to the plastid by Deeploc. DAPs are red and non-significant proteins are blue.

Given the massive shift in redox stoichiometry of the *K. brevis* proteome, we explored DAPs that were more abundant in *Kb*HT for proteins related to antioxidant defense. A volcano plot that includes 52 antioxidant defense-related enzymes that were identified in our dataset is shown in Figure 6A. Among these enzymes, 32 were DAPs and 25 of the DAPs were higher in *Kb*HT. This included the iron-dependent alternative oxidase (AOX), which was an astonishing 17-fold more abundant in *Kb*HT. An ABC-type Fe^+3^ transporter was 15-fold more abundant in *Kb*HT, presumably transporting iron for increased AOX biosynthesis. Furthermore, an ATP synthase was also 17-fold higher in *Kb*HT. Along with the AOX, and shown in Figure 6A, 24 other proteins related to antioxidant defense were significantly more abundant in *Kb*HT. The enzymes represented in Figure 6A include ascorbate peroxidase (APx); alkyl hydroperoxide reductase (AhpC) (both DAPs and 7.5- and 6-fold increase in *Kb*HT); multiple isoforms of thioredoxin; thioredoxin reductase; superoxide dismutase; glutaredoxin (Grx); peroxiredoxin (Prx); glutathione-S-transferase (GST); glutathione peroxidase (GPx); several enzymes simply annotated as “peroxidase”; as well as enzymes related to glutathione biosynthesis, i.e., lactoyl glutathione lyase, hydroxyacyl glutathione hydrolase, and S-formyl glutathione hydrolase.

The Fisher’s exact test revealed significant associations with six cellular compartments based on DeepLoc localizations: plastid (enrichment *Kb*HT/*Kb*LT = 1.54, *p-*value = 2.8 × 10^−26^, FDR = 1.42 × 10^−24^), extracellular (enrichment *Kb*HT/*Kb*LT = 1.35, *p-*value = 6.8 × 10^−21^, FDR = 1.71 × 10^−19^), Golgi apparatus (enrichment *Kb*HT/*Kb*LT = 1.30, *p-*value = 2.0 × 10^−4^, FDR = 1.74 × 10^−3^), cytoplasm (enrichment *Kb*HT/*Kb*LT = 0.92, *p-*value = 1.35 × 10^−5^, FDR = 1.68 × 10^−4^), mitochondrian (enrichment *Kb*HT/*Kb*LT = 0.85, *p-*value = 1.95 × 10^-3^, FDR = 1.40 × 10^−3^), and endoplasmic reticulum (ER, enrichment *Kb*HT/*Kb*LT = 0.74, *p-*value = 1.37 × 10^−5^, FDR = 1.33 × 10^−3^). Enrichment factors are indicative of more uniformity between strains in the cytoplasm, mitochondrion, and ER and increased variability in the plastid, Golgi apparatus, and extracellular structures. A sixth group categorized as either cytoplasm or nucleus was also significantly different (enrichment *Kb*HT/*Kb*LT = 0.70, *p-*value = 1.36 × 10^−9^, FDR = 2.28 × 10^−8^). As nuclear proteins were not significantly different, this was likely due to cytoplasmic proteins also classified in this group. A volcano plot and heat map of plastid proteins are shown in Figure 6B,C. The 1 D annotation enrichment feature in Perseus determined that plastid-localized proteins were higher in all four *Kb*HT samples (*p-*values < 1.5 × 10^−25^, FDR < 7.6 E -24) and lower in all five *Kb*LT samples (*p-*values < 1.59 × 10^−31^, FDR < 9.5 × 10^−30^).

## 3. Discussion

Two long-standing questions regarding dinoflagellates polyether biosynthesis are why and how these energetically costly molecules are produced. Considering the resources committed to their construction, one would presume that some advantage is conferred by their presence. Using fluorescent and photoaffinity probes, brevetoxin was localized to the chloroplasts of *K. brevis* and was found to interact with the light harvesting complex and thioredoxin [18], a key component of the cellular antioxidant defense system. The thioredoxin system is composed of thioredoxin (Trx) and thioredoxin reductase (TrxR). Together, these enzymes function to maintain redox homeostasis with a cell, and they are essential components of the antioxidant defense system. Trx regulates the activity of numerous target proteins by reducing disulfide bridges by a thiol–disulfide exchange. In the process, the catalytic Trx dithiol is oxidized to the disulfide. TrxR returns Trx to its active, reduced state using NADPH [58]. Brevetoxin (PbTx-2) has been shown to be an inhibitor of recombinant *K. brevis* thioredoxin reductase (*Kb*TrxR) [19]. The IC_50_ value for inhibition of *Kb*TrxR by PbTx-2 is modest (56 μM), but potentially impactful, given that intracellular levels of brevetoxins can reach millimolar concentrations. We reasoned that the inhibition of *Kb*TrxR by PbTx-2 should impact the redox state of the *K. brevis* proteome. Indeed, using iodo-TMT labeling, we observed a broad, significant shift toward oxidation in the high-toxin strain (*Kb*HT), spanning multiple pathways, enzyme classes, and organelles, representing a key finding of this study.

These observations lead to the conspicuous question: what advantage, if any, does the production of PbTx-2 provide to *Kb*HT? The inhibition of TrxR can have profound impacts on cellular processes as it regulates the activity of numerous enzymes through its principal substrate Trx. Photosynthetic organisms, including *K. brevis*, express multiple isoforms of Trx, and not all Trx targets have been identified even in the most well-studied plants [59,60], which presents a challenge to assessing the full breadth of this effect. We have previously described how the shift to a more reduced proteome in *Kb*LT can negatively influence processes whose enzymes require intramolecular disulfide bridges or nucleophilic cysteine residues to maintain full catalytic activity [46]. On the other hand, PbTx-2 may negatively impact the thioredoxin system of competitors. *K. brevis* has been shown to induce oxidative stress in the diatom *Thalassiosira pseudonana* [15,16]. In addition *Karenia mikimotoi*, which produces the structurally similar polyether ladder gymnocin, has been shown to induce oxidative stress in the marine copepod *Tigriopis japonicus* [61]. Deng and co-workers demonstrated that bacteria that inhabit the phycopshere of toxic dinoflagellates, including that of *K. brevis*, tend to be enriched in genes coding for antioxidant defense-related enzymes [62].

We propose that the inhibition of *Kb*TrxR by PbTx-2 leads in turn to the disruption of redox homeostasis, promoting oxidative stress. *Kb*HT may compensate for TrxR inhibition by increasing the production of enzymes related to antioxidant defense such as the AOX, APx, AhpC, Grx, GST, SOD, GPx, and Prx. *Kb*HT has previously been shown to have lower levels of ascorbic acid, reduced glutathione (GSH), and high molecular weight thiols compared to *Kb*LT. Yet, *Kb*HT showed an overall higher antioxidant capacity [46,63]. Interestingly, we showed a slightly higher Trx and Grpx activity in *Kb*LT [63], which is not consistent with our current findings of enrichment of these enzymes in *Kb*HT. On the other hand, the activity of Trx and Grx depend on active site cysteines, which, when oxidized, become inactivated [58,64]. Therefore, these enzymes may need to be enriched under highly oxidizing conditions to achieve the same activity. The type of excess cysteine oxidation in *Kb*HT proteins cannot be determined from our data. Reversible cysteine oxidations include sulfenic acids, S-nitrosocysteines, protein disulfide bridges, or glutathionylated cysteines. The lower levels of GSH and upregulation of GST may indicate high levels of protein glutathionylation as a mechanism to protect against brevetoxin-induced oxidative stress.

The AOX is a member of a non-heme diiron carboxylate family of proteins and an alternative pathway of mitochondrial electron transport [65]. The AOX pathway is uncoupled from ATP production as it transfers electrons directly from ubiquinol to reduce oxygen to water without contributing to the proton gradient that drives ATP production. The AOX is believed to contribute to biotic and abiotic stress tolerance in plants, algae fungi, and animals and to suppress excessive reactive oxygen species (ROS) generation in both mitochondria and chloroplasts [66,67,68,69]. ATP regulates the activity of AOX via a negative feedback mechanism. In the parasitic protist *Trypanosoma brucei*, an ATP synthase was found to operate in reverse mode [70]. That is, it functions as an ATPase with H+ pump activity, reducing cellular ATP concentration. The increase in abundance of an ATP synthase and an Fe^3+^ transporter may support enhanced AOX biosynthesis and activity.

Recently, there has been significant interest and effort focused on red tide suppression or mitigation, including the Florida red tide [71,72]. An intricate understanding of the unique biochemistry of these harmful algae would support these efforts. The AOX is common in protists, and is, in fact, the terminal oxidase of the Trypanosome parasite *Trypanosoma brucei brucei*, which is responsible for African sleeping sickness. As such, it has been identified as a drug target for treatment of the disease, and numerous AOX inhibitors have been discovered [65], including ascofuranone, a fungal-derived terpene. Our discovery of enhanced AOX production suggests that *Kb*HT is reacting to the more oxidized redox state of the cell associated with the presence of brevetoxins, and as such, may be more sensitive to AOX inhibitors than *Kb*LT. This discovery offers a novel approach to the mitigation of the Florida red tide by selectively targeting high toxin-producing strains with AOX inhibitors or the appropriate fungi.

Polyketides are constructed from the sequential condensation of carboxylic acid units, extending the growing carbon chain by two carbons at time. Polyketide synthases can be composed of multienzyme complexes that act on growing polyketides by passing intermediate substrates between individual enzymes. On the other hand, large multidomain/multimodular enzymes pass the growing intermediates along sequential modules in an assembly line fashion. The products of multimodular PKSs can often be predicted based on module order. Conversely, the sequence of modules may be predicted by inspection of the polyketide structure. Recently, two multimodular gigasynthases have been identified in the haptophyte algae *Prymnesium parvum* [73]. The 56 modules of PKZILLA-1 and-2 align with the predicted architecture and are believed to be responsible for the construction of the ladder frame polyether prymnesins. The size and complex structure of the dinoflagellate-derived ladder frame polyethers suggest that they may also express similar giant PKSs. Early studies in dinoflagellates identified numerous transcripts encoding single-function PKS enzymes. More recently, multidomain/multimodular transcripts have been identified in *K. brevis*, *Symbiodinium minutim*, and in the ciguatoxin-producing *G. polynesiensis* [34,40,74]. None of these, however, are comparable to the size of the PKZILLAs or can fully account for the biosynthesis of brevetoxins or ciguatoxins. Our custom proteome was derived from the translation of transcriptome libraries generated using short-read sequencing [56]. As a result, it does not contain any large multidomain PKSs comparable to the PKZILLAs, and thus such enzymes could not have been detected in our dataset. However, numerous single-domain or small multidomain PKSs from *K. brevis* were significantly more abundant in KbHT. These enzymes alone would not be sufficient for complete brevetoxin biosynthesis as proposed by the PKZILLA model. This suggests that, unlike prymnesins, brevetoxins may be synthesized by a complex of interacting single- or small multidomain PKS enzymes. Alternatively, the single- and small multidomain PKSs identified may be fragments of larger, multidomain enzymes, highlighting the difficulty of reconstructing long, repetitive transcripts from short-read sequencing data.

The identification and analysis of the PKZILLAs were facilitated by the mining of a complete *P. parvum* genome assembly where PKZILLAs were found. Unfortunately, even a draft genome of *K. brevis* is unlikely to appear soon and predicting how multiple PKS enzymes interact to coordinate the assembly of large polyketide products is not possible using transcriptomics alone. The differentially abundant PKSs identified in this work represent excellent leads for pursuing the biosynthetic pathway for the brevetoxins or other *K. brevis*-derived polyketides at the protein level. Affinity enrichment or pulldown experiments may allow us to piece together the enzymes responsible for brevetoxin biosynthesis.

Proteomic studies of *K. brevis* are severely limited by the lack of a reference genome, or an annotated reference proteome, and we fully acknowledge this limitation. At the time of this writing, 71,931 *K. brevis* proteins can be found in the NCBI database and 68,945 are described as “unnamed protein”. Lacking a reference proteome, reliable statistical analysis by categorical groupings is difficult if not impossible. Fewer than half of the proteins that appear in our dataset could be annotated, and our limited annotation of the proteome was based on in silico comparisons. Highly conserved proteins are likely to be annotated correctly. Arguably, less-conserved proteins may be annotated incorrectly, and enzymes with highly conserved domains could be misidentified. For example, proteins annotated as glutaredoxin or protein disulfide isomerase (PDI) might in fact be Trx, both of which contain a highly conserved thioredoxin-fold [75] and function by thiol–disulfide exchange. The assessment of pathway enrichment applies statistical tests that rely on the definition of background genes or proteins for comparison. All annotated proteins or genes are often used as the default; however, when background data includes only a subset of all proteins, the inflation of *p-*values may result in false-positive identifications. To complicate matters further, each of the proteins potentially can have multiple annotation terms. The choice of terms to be used in the enrichment analysis can significantly shift the outcome and is not a trivial decision. Nonetheless, we are confident in our PKS annotations, and the 1D enrichment analysis relies only on accurate annotation of the specific group in question and not necessarily accurate annotation of background proteins.

Our *K. brevis* cultures are unialgal but not axenic: they do contain associated bacteria. Nonetheless, the analysis performed by us and others [34] lend confidence that the identified PKSs originate from *K. brevis* and not from bacteria. Distinct bacterial communities have been reported in toxic and non-toxic dinoflagellate strains [62], and others have reported stimulation or suppression of toxins by associated bacteria [76]. However, we have not yet assessed *Kb*HT and *Kb*LT for differences in the associated bacterial communities.

While there are limitations to what we can currently learn from proteomics experiments on non-model organisms, the demonstration of a massive shift in the redox state of the proteome and the large percentage of DAPs between high- and low-toxin strains of *K. brevis* do not require a fully annotated proteome to be meaningful. These findings highlight the profound biochemical impact of brevetoxins and suggest an important functional role. The identification of differentially abundant PKSs offers valuable leads for probing the biosynthesis, while the enrichment of antioxidant defense proteins aligns with redox trends and previous strain comparisons, pointing to a potential strategy for selectively targeting high-toxin producers.

## 4. Materials and Methods

### 4.1. K. brevis Culture

Cultures of high and low toxin-producing *K. brevis* (Wilson strains) were obtained from Mote Marine Laboratory (Sarasota, FL, USA) and maintained in the L1-Si medium [77], with the exception that the NH 15 vitamin supplement [78] replaced the L-1 vitamin supplement. Cultures were diluted to 10,000 cells/mL and were maintained in a Percival growth chamber (Model I-66LLVL) at 25 °C using a 12:12 day/night cycle at 25% of full light intensity. Growth was monitored by counting cells using a Countess 3FL cell counter with a Cy5 2.0 light cube. Cells were harvested on days 13 and 17 post dilution, 4 h after the start of the daylight phase. Cell counts and growth curves are provided in the Supplementary Materials.

### 4.2. Brevetoxin Extraction and Liquid Chromatography–Mass Spectrometry (LC–MS) Analysis

*K. brevis* culture in the logarithmic growth phase was diluted to 800,000 cells/Liter in filtered, ozonated seawater. Brevetoxins were extracted from subsamples according to previously described methods [71]. Briefly, 50 mL aliquots of diluted culture were passed through Strata C-18-E cartridges (Phenomenex, Torrance, CA, USA) using a PromoChrom SPE-O3 automated sample preparation system (PromoChrom, Cincinnati, OH, USA) and analytes were eluted with LCMS-grade methanol. Extracts were concentrated by evaporation under ultra-high purity nitrogen stream at 40 °C, reconstituted in 100% LCMS-grade methanol, and syringe-filtered using 0.2 um polytetrafluoroethylene (PTFE) chromatography syringe filters (Cole-Palmer, Vernon Hills, IL, USA) into glass autosampler vials. Extracts were stored at −20 °C until analysis. Brevetoxin congeners (BTX-1, BTX-2, and BTX-3) were quantified by HPLC-MS/MS analysis using a Vanquish HPLC system coupled to a TSQ Quantis triple quadrupole mass spectrometer equipped with an electrospray interface (LC/ESI/MS/MS) (Thermo Fisher Scientific Inc., Waltham, MA, USA). Reference standards for BTX-1, BTX-2, and BTX-3 (MARBIONC University of North Carolina, Wilmington, NC, USA) were used for instrument calibration and method quality assurance. The results of the toxin analysis are included in the Supplementary Materials (Table S2).

### 4.3. Peptide Isolation and Labeling

Cells were harvested in 50 mL falcon tubes by the centrifugation of culture at 800× *g* for 10 min. Protein isolation, digestion, and labeling with iodo-TMT reagents were previously described [46]. Briefly, four biological replicate samples of *Kb*HT and *Kb*LT were prepared according to the workflow in Figure 2B. Proteins were labeled before and again after the reduction of oxidized cysteines with TCEP using TMT reagents having a different isotope distribution across the reporter and equalizer. Peptides for amine reactive TMT labeling were prepared using the EasyPep MS sample prep kit according to the manufacturer’s instructions. Five replicates each of *Kb*HT and *Kb*LT were prepared. Pellets were resuspended in 100 μL of the provided lysis buffer. Proteins were quantified prior to digestion using Bradford reagent against a standard curve of BSA. Typical protein concentrations were in the range of 0.5–1 mg/mL, and 100 μg of protein from each sample was digested. A reference channel consisted of a pooled sample of each (100 µg total or 10 μg each) of the ten samples. Peptides were quantified after digestion using the Pierce Quantitative Colorimetric Peptide Assay kit according to the manufacturer’s instructions, and 70 μg of each peptide sample was labeled. Peptides were labeled with amine reactive TMT reagents using the TMT10plex Isobaric Label Reagent Set plus TMT-131C for the reference channel. *Kb*HT was labeled with 126, 127N, 127C, 128N, and 129N. *Kb*LT was labeled with 128C, 129C, 130C, 130N, and 131N. After labeling, samples were pooled into a single sample, and the pooled sample was separated into eight fractions using the Pierce High pH Reversed-Phase Peptide Fractionation kit.

### 4.4. Liquid Chromatography–Mass Spectrometry (LC–MS) Analysis of Labeled Peptide Samples

Samples were reconstituted in a 2% acetonitrile, 0.1% TFA buffer and diluted such that ~1 μg of peptides was injected per sample or per fraction. Peptides were analyzed on a Thermo Orbitrap Eclipse MS system coupled to an Ultimate 3000 RSLC-Nano liquid chromatography system. Samples were injected onto a 75 um i.d., 75 cm long EasySpray column (Thermo) and eluted with a gradient (from 1 to 28% buffer B over 180 min and from 28 to 45% buffer B in 25 min for iodo-TMT-labeled samples or from 0 to 28% buffer B for TMT-labeled samples), at a flow rate of 250 nL/min. Buffer A contained 2% (*v*/*v*) ACN and 0.1% formic acid in water, and buffer B contained 80% (*v*/*v*) ACN, 10% (*v*/*v*) trifluoroethanol, and 0.1% formic acid in water. Spectra were continuously acquired in a data-dependent manner throughout the gradient, acquiring a full scan in the Orbitrap (at 120,000 resolution with a standard AGC target), followed by MS/MS scans on the most abundant ions in 2.5 s in the ion trap (turbo scan type with an intensity threshold of 5000, CID collision energy of 35%, standard AGC target, maximum injection time of 35 ms, and isolation width of 0.7 *m*/*z*). Charge states from 2 to 6 were included. Dynamic exclusion was enabled with a repeat count of 1 and an exclusion duration of 25 s and an exclusion mass width of ±10 ppm. A real-time search was used for the selection of up to 10 MS2 peaks for the SPS-MS3 analysis, in the Orbitrap at a resolution of 50,000, HCD collision energy of 65% (TMT-labeled) or a resolution of 30,000, HCD collision energy of 58% (iodo-TMT labeled), and a scan range of 100–500.

Raw MS data files from the eight TMT fractions were merged, and protein identification and quantification were completed using Proteome Discoverer v.3.0 (Thermo) for TMT samples or v.2.4 (Thermo) for iodo-TMT samples, analyzed against the *K. brevis* translated transcriptome database [56]. Both Comet and SequestHT with INFERYS Rescoring were used. The carbamidomethylation (+57.0215) of cysteine and TMT reagent (+229.1629) of lysine and peptide N-termini or iodo-TMT (+229.1629) of cysteine were used as static modifications, and the oxidation (+15.9949) of methionine was used as a variable modification for both sets of labeled peptides. Up to 2 (TMT-labeled) or 3 (iodo-TMT-labeled) missed tryptic cleavage were allowed.

### 4.5. Functional Annotation of the Identified Proteins

Protein sequences derived from the six-frame translation of the *K. brevis* Wilson strain transcriptome library [56] with stop codons removed were annotated by CD Genomics using the eggNOG mapper tool [79]. From the transcriptome library containing over 80,000 sequences, 519,481 translated protein sequences were obtained, of which 11,487 could be annotated. Among the 5044 proteins identified in our dataset, 1655 were annotated using the eggNOG mapper, and another 434 were annotated by submitting the protein sequence to NCBI BLASTp against the SAR (stramenopiles, alveolates, and rhizarians; taxid:2698737) supergroup [57]. Annotations were made when a protein was identified in three or more genera, with an E-value of < 10^−9^ and >50% query coverage. In total, 2089 sequences were annotated, while 1941 were assigned to a COG classification, 1635 to a Pfam classification, 1200 were assigned a KO term, and 948 were assigned to a KEGG pathway. To this database were added translations of all *K. brevis* PKS transcripts reported by Van Dolah [34].

### 4.6. Analysis of Proteomic Data

For the iodo-TMT samples, the redox stoichiometry for each protein was calculated by dividing the summed reporter ion intensities for the light label of each protein (reduced cysteines) by the sum of reporter ion intensities of both labels (total cysteines) for each individual protein. Redox stoichiometry values were used as input for Perseus. The fold change in redox stoichiometry was expressed as KbHT/*Kb*LT. *p*-values were calculated using Student’s *t*-test in Excel (two-tailed, homoscedastic) and in Perseus.

For TMT samples, reporter ion intensities for all peptides matched to each respective protein were summed to create total protein intensities. The intensities of 18 proteins that were identified as chlorophyll A/B binding protein (LHCII) were summed to a single line prior to analysis. Either due to low protein yield or inefficient labeling, one channel (129N *Kb*HT) had a summed reporter intensity that was two orders of magnitude lower than the other channels, with more than half of the proteins missing. This channel was dropped from the analysis. Reporter intensities for the remaining channels were multiplied by global scaling factors specific to each channel such that all total channel intensities were equal.

Scaled reporter intensities served as input for Perseus. Intensities were log_2_ transformed and normalized by subtraction of the mean. Similar results were obtained by subtraction of the median, but the results are reported here using mean normalization. The differential abundance was calculated as the difference of the average log_2_ protein intensities of *Kb*HT-*Kb*LT. *p-*values were calculated using the *t*-test in Perseus and in Excel (two-tailed, homoscedastic) using the method described by Aguilan [80]. Heat maps were produced using Perseus after Z-score normalization of the protein dataset, and minimum and maximum intensity values were represented by default green and red colors, respectively. The 1D annotation enrichment analysis and Fisher’s exact tests were performed in Perseus applying a Benjamini–Hochberg FDR threshold value of 0.02.

## Data Availability

The mass spectrometry proteomics data have been deposited to the ProteomeXchange Consortium via the MassIVE partner repository and are publicly available as of the date of publication. Iodo-TMT samples: Accession number: MSV0000095971. TMT11plex samples: Accession number: MSV0000095972.

## Supplementary Materials

The following supporting information can be downloaded at: https://www.mdpi.com/article/10.3390/md23070291/s1, Supplemental File S1: Cell counts, growth curves, toxin analysis. Supplemental File S2: *K. brevis* translated transcriptome .txt file (519,408 proteins). Supplemental File S3: *K. brevis* annotated proteins .txt file (14,859 proteins). Supplemental File S4: Perseus output that includes protein identification (Locus within the transcriptome library), DeepLoc localization, COG class, protein description, InterProScan, normalized TMT intensities, fold change, *p*-value (two-tailed, homoscedastic, unpaired *t*-test) (5044 proteins). Supplemental File S5: Secondary metabolite heat map with names.
