# Supplementary material for: Comparative Quantitative Proteomic Analysis of High and Low Toxin-Producing Karenia brevis Strains Reveals Differences in Polyketide Synthase Abundance and Redox Status of the Proteome
Source: Mar Drugs. 2025 Jul 17;23(7):291. doi: 10.3390/md23070291 (PMC12300183; doi:10.3390/md23070291)
Supplement: Supplementary file 1 [file marinedrugs-23-00291-s001.zip › Supplemental S1_Growth Curves_Toxin Analysis.pdf]

Supplementary Materials

Comparative quantitative proteomic analysis of high and low toxin-producing *Karenia brevis* strains reveals differences in polyketide synthase abundance and redox status of the proteome

Kathleen Rein, Ricardo Colon, Carlos Romagosa, Nick Ohnikian, Kirstie Francis, Samuel Rein.

Table S1. K. brevis cell counts for cultures used for amine reactive TMT labeling

	Cell counts		St. Dev	
Day	*Kb*HT	*Kb*LT	*Kb*HT	*Kb*LT
0	10000	10000	1250	1182
2	11131	9250	1183	2045
4	15031	12527	1503	4672
7	21218	15197	1998	8926
9	27349	15047	5725	7723
11	34887	19853	4227	8527
13	38636	29862	5782	7735
17	42555	34027	7283	8846
21	48327	42128	7803	12155
24	40033	46182	11205	15379

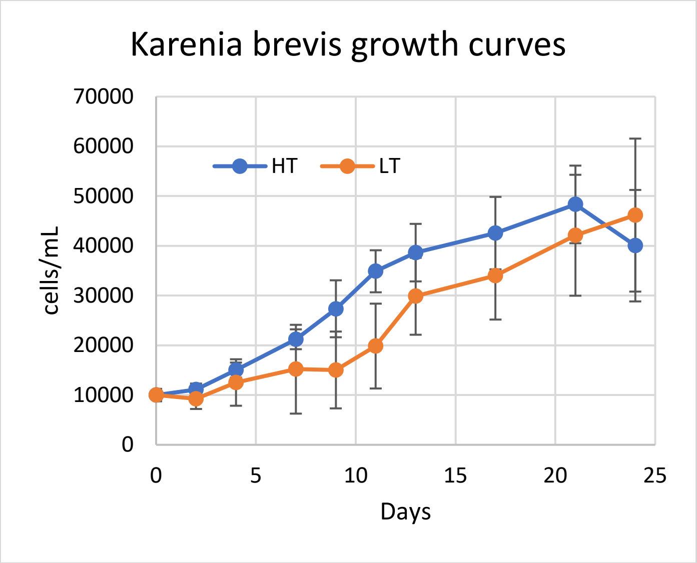

Figure S1. K. brevis growth curves for cultures used for amine reactive TMT labeling

Table S2. *K. brevis* culture: Toxin concentrations. The data presented are the results of analysis corrected for any amounts lost in the extraction process by using the standard recovery samples which were processed with the sample batch.

Sample ID	Sample Name	*K. brevis* strain	Sample Date	Corrected PbTx-1 ng/L LoQ 243 ng/L	Corrected PbTx-2 ng/L LoQ 126 ng/L	Corrected PbTx-3 ng/L LoQ 94 ng/L
R24-2095	WIL-86_1	*Kb*HT	5/14/2024	440	3600	120
R24-2096	WIL-86_2	*Kb*HT	5/14/2024	390	3500	100
R24-2097	WIL-86_3	*Kb*HT	5/14/2024	400	3400	100
R24-2098	WLT-28_1	*Kb*LT	5/14/2024	0	bpql*	0
R24-2099	WLT-28_2	*Kb*LT	5/14/2024	0	bpql*	0
R24-2100	WLT-28_3	*Kb*LT	5/14/2024	0	0	0
R24-2106	WIL-87_1	*Kb*HT	5/21/2024	360	3100	bpql*
R24-2107	WIL-87_2	*Kb*HT	5/21/2024	480	3400	bpql*
R24-2108	WIL-87_3	*Kb*HT	5/21/2024	480	3500	bpql*
R24-2109	WLT-29_1	*Kb*LT	5/21/2024	0	0	0
R24-2110	WLT-29_2	*Kb*LT	5/21/2024	0	0	0
R24-2111	WLT-29_3	*Kb*LT	5/21/2024	0	0	0
R24-2117	WIL-88_1	*Kb*HT	5/28/2024	bpql*	1700	bpql*
R24-2118	WIL-88_2	*Kb*HT	5/28/2024	280	1800	bpql*
R24-2119	WIL-88_3	*Kb*HT	5/28/2024	280	1700	bpql*
R24-2120	WLT-30_1	*Kb*LT	5/28/2024	0	0	0
R24-2121	WLT-30_2	*Kb*LT	5/28/2024	0	0	0
R24-2122	WLT-30_3	*Kb*LT	5/28/2024	0	0	0

***bpql**=below practical quantitation limit (see LoQ) In these cases there is a peak and confirming fragment which indicates the presence of the compound but is below the calibration range of the method.
