# Supplementary figures and images for: Comparative Quantitative Proteomic Analysis of High and Low Toxin-Producing Karenia brevis Strains Reveals Differences in Polyketide Synthase Abundance and Redox Status of the Proteome
Source: Mar Drugs. 2025 Jul 17;23(7):291. doi: 10.3390/md23070291 (PMC12300183; doi:10.3390/md23070291)

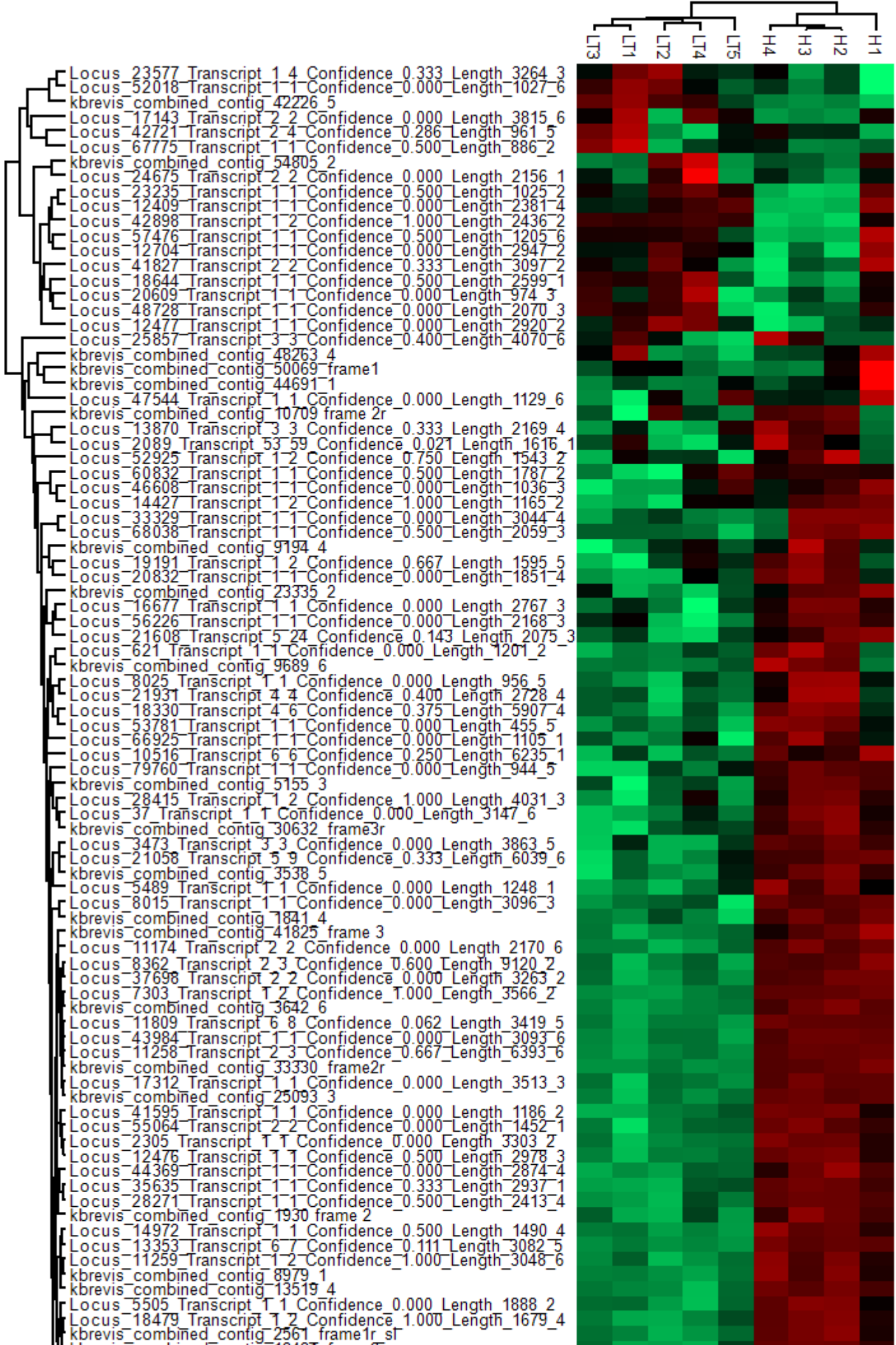

Supplement: Supplementary file 1 [file marinedrugs-23-00291-s001.zip › Supplemental file S5 Secondary metabolite heat map with names.pdf]
